# Supplementary material for: From sequence to enzyme mechanism using multi-label machine learning
Source: BMC Bioinformatics. 2014 May 19;15:150. doi: 10.1186/1471-2105-15-150 (PMC4229970; doi:10.1186/1471-2105-15-150)
Supplement: Additional file 2 — Java code of ml2db. Additional file ml2db_code.tar.gz contains the Java source code to run the multi-label machine learning experiments and save the results to database. The code’s Javadoc is included. [file 1471-2105-15-150-S2.zip › additional file 2/ml2db/ml2db/doc/mulan/evaluation/measure/MacroAverageMeasure.html]

MacroAverageMeasure


JavaScript is disabled on your browser.


- Overview
- Package
- Class
- Use
- Tree
- Deprecated
- Index
- Help

- Prev Class
- Next Class

- Frames
- No Frames

- All Classes

- Summary:
- Nested |
- Field |
- Constr |
- Method

- Detail:
- Field |
- Constr |
- Method


mulan.evaluation.measure

## Interface MacroAverageMeasure

- All Known Implementing Classes:
  :   GeometricMeanAverageInterpolatedPrecision, GeometricMeanAveragePrecision, MacroAUC, MacroFMeasure, MacroPrecision, MacroRecall, MacroSpecificity, MeanAverageInterpolatedPrecision, MeanAveragePrecision

  ---

    

  ```
  public interface MacroAverageMeasure
  ```

  Interface for macro average measures

  Version:
  :   2012.07.27

  Author:
  :   Grigorios Tsoumakas

- - ### Method Summary

    Methods

    | Modifier and Type | Method and Description |
    | --- | --- |
    | `double` | `getValue(int labelIndex)` Returns the value of a macro average measure for a particular label |

- - ### Method Detail


    - #### getValue

      ```
      double getValue(int labelIndex)
      ```

      Returns the value of a macro average measure for a particular label

      Parameters:
      :   `labelIndex` - the index of the label [0..numLabels-1]

      Returns:
      :   the value of a macro average measure for a particular label


- Overview
- Package
- Class
- Use
- Tree
- Deprecated
- Index
- Help

- Prev Class
- Next Class

- Frames
- No Frames

- All Classes

- Summary:
- Nested |
- Field |
- Constr |
- Method

- Detail:
- Field |
- Constr |
- Method
